# Association between Height and Functional Outcomes of Patients 6 Months after a Stroke: A Multicenter Prospective Observational Cohort Study

**DOI:** 10.3390/jpm14060610

**Published:** 2024-06-07

**Authors:** Nae Yoon Kang, Sung-Hwa Ko, Yong-Il Shin, Ji Hong Min, Mi Sook Yun, Min Kyun Sohn, Jongmin Lee, Deog Young Kim, Gyung-Jae Oh, Yang Soo Lee, Min Cheol Joo, So Young Lee, Min-Keun Song, Junhee Han, Jeonghoon Ahn, Yun-Hee Kim, Won Hyuk Chang

**Affiliations:** 1 Department of Rehabilitation Medicine, School of Medicine, Pusan National University, Pusan National University Yangsan Hospital, Yangsan 50612, Republic of Korea; dr.knybs12@gmail.com (N.Y.K.); rmshin01@gmail.com (Y.-I.S.); papered@hanmail.net (J.H.M.); 2 Research Institute for Convergence of Biomedical Science and Technology, Pusan National University Yangsan Hospital, Yangsan 50612, Republic of Korea; 3 Division of Biostatistics, Research Institute for Convergence of Biomedical Science and Technology, Pusan National University Yangsan Hospital, Yangsan 50612, Republic of Korea; msyun@pusan.ac.kr; 4 Department of Rehabilitation Medicine, School of Medicine, Chungnam National University, Daejeon 35015, Republic of Korea; mksohn@cnu.ac.kr; 5 Department of Rehabilitation Medicine, School of Medicine, Konkuk University, Seoul 05029, Republic of Korea; leej@kuh.ac.kr; 6 Department and Research Institute of Rehabilitation Medicine, College of Medicine, Yonsei University, Seoul 03722, Republic of Korea; kimdy@yuhs.ac; 7 Department of Preventive Medicine, School of Medicine, Wonkwang University, Iksan 54538, Republic of Korea; pmokj@wku.ac.kr; 8 Department of Rehabilitation Medicine, School of Medicine, Kyungpook National University, Kyungpook National University Hospital, Daegu 41944, Republic of Korea; leeyangsoo@knu.ac.kr; 9 Department of Rehabilitation Medicine, School of Medicine, Wonkwang University, Iksan 54538, Republic of Korea; jmc77@hanmail.net; 10 Department of Rehabilitation Medicine, School of Medicine, Jeju National University, Jeju 63241, Republic of Korea; bluelsy900@hanmail.net; 11 Department of Physical and Rehabilitation Medicine, Chonnam National University Medical School, Gwangju 61469, Republic of Korea; drsongmk@daum.net; 12 Department of Statistics, Hallym University, Chuncheon 24252, Republic of Korea; pnuyh.rass@gmail.com; 13 Department of Health Convergence, Ewha Womans University, Seoul 03760, Republic of Korea; ahnjeonghoon@ewha.ac.kr; 14 Department of Physical and Rehabilitation Medicine, Center for Prevention and Rehabilitation, Heart Vascular and Stroke Institute, Samsung Medical Center, School of Medicine, Sungkyunkwan University, Seoul 06351, Republic of Korea; yun1225.kim@samsung.com

**Keywords:** post-stroke recovery, functional outcomes, height, stroke rehabilitation, disability, activities of daily living

## Abstract

Many physical factors influence post-stroke functional outcomes. However, few studies have examined the influence of height on these outcomes. Here, data from the Korean Stroke Cohort for Functioning and Rehabilitation were used and patients’ height was categorized into three groups: short (lower 25%), middle (middle 50%), and tall (upper 25%). Differences in the modified Rankin scale (mRS), functional ambulatory category (FAC), and Korean-translated version of the Modified Barthel Index (K-MBI) scores were analyzed for each group at 6 months post-stroke. A subgroup analysis was conducted based on the initial Fugl-Meyer Assessment (FMA) score. We analyzed functional outcomes in 5296 patients at 6 months post-stroke, adjusting for age and body mass index. The short-height group exhibited higher mRS scores (1.88 ± 0.043), lower FAC scores (3.74 ± 0.045), and lower K-MBI scores (82.83 ± 0.748) than the other height groups (*p* < 0.05). In the subgroup analysis, except for the very severe FMA group, the short-height group also exhibited worse outcomes in terms of mRS, FAC, and K-MBI scores (*p* < 0.05). Taken together, the short-height group exhibited worse outcomes related to disability, gait function, and ADLs at 6 months post-stroke.

## 1. Introduction

Stroke is a major disease resulting in long-term disabilities [1]. From 1990 to 2019, age-standardized rates of stroke incidence and mortality decreased by 17% and 36%, respectively. However, the absolute number of incident strokes increased by 70% and disability-adjusted life-years (DALYs) due to stroke increased by 32% during the same period [2]. In South Korea, the incidence rate of stroke in 2019 increased by 31.1% compared to 2012, whereas the number of deaths due to stroke decreased from 26,514 in 2010 to 21,586 in 2019 [3]. Consequently, more people are living with post-stroke disabilities, increasing the burden on individuals and society [4]. Functional improvement through effective stroke rehabilitation is essential to reduce healthcare costs associated with post-stroke disability [5].

The clinical presentation of patients with stroke is diverse, and each individual has a unique recovery pattern and expectations based on their physical characteristics [6]. It is crucial to understand the influence of physical factors on functional outcomes after stroke, as these factors are easily observable in clinical settings. This understanding allows for individualized treatment intensity and appropriate goal-setting to enhance rehabilitation efficiency.

Many physical factors have been reported to influence post-stroke functional outcomes [7]. Among these, age, body mass index (BMI), and sex, categorized as non-modifiable factors, have been extensively studied [8,9,10,11]. A younger age at stroke onset is associated with better outcomes in terms of disability and activities of daily living (ADLs) at 6 months post-stroke [8]. BMI shows a positive correlation with ADLs at 6 months post-stroke [9,10]. Female patients with stroke tend to have poorer functional recovery compared to males [11].

However, few studies have examined the impact of height, a common and easily measurable anthropometric factor, on functional outcomes after stroke. One study exists, but it found that height at hospital admission had no effect on discharge outcome [12]. There are a few studies that have analyzed the association of height with medical characteristics and physical functioning in the general population, but they tend to be inconsistent. Regarding research on medical characteristics, a short height has been associated with high stroke mortality and previous stroke incidence [12,13,14]. Taller individuals have increased cancer risk and mortality [15,16]. Moreover, some reports have suggested a correlation between taller height and an increased risk of atrial fibrillation due to larger atrial dimensions [17,18]. In terms of body function, taller height is also linked to better maximal aerobic exercise in healthy young populations [19]. Conversely, others have stated that in athletes, height is not significantly associated with exercise performance [20]. In addition, taller height has been associated with decreased balance due to a higher center of gravity (COG) [21].

Regarding height and balance after a stroke, patients tend to bear most of their weight on the non-paralyzed leg, decreasing balance and posture control [22,23]. Combined with height-related balance impairment and post-stroke balance decrease, it is generally thought that taller patients with stroke are expected to exhibit more pronounced functional impairments associated with balance. Thus, we hypothesized that height is associated with functional outcomes in patients with stroke.

This study aimed to explore the relationship between height and functional outcomes in patients with chronic stroke. Specifically, we aimed to determine how height influences functional outcomes and whether this relationship varies based on sex and the initial motor impairment severity. Identifying this relationship may help predict outcomes in post-stroke rehabilitation.

## 2. Materials and Methods

### 2.1. Study Population

This report is part of the Korean Stroke Cohort for Functioning and Rehabilitation (KOSCO) study, a multicenter prospective observational cohort study. The KOSCO study was designed as a 10-year, long-term follow-up of patients with stroke to investigate residual disabilities, activity limitations, and other health-related outcomes. The detailed rationale and protocols of the KOSCO study were described previously [24]. The study recruited all patients admitted to nine representative hospitals in Korea with a diagnosis of acute first-ever stroke from July 2012 to May 2015. Inclusion criteria were as follows: (1) first-ever acute stroke (ischemic or hemorrhagic) with a corresponding lesion and/or evidence of acute arterial occlusion on computed tomography (angiography) or magnetic resonance imaging/angiography scan, (2) age ≥ 19 years at stroke onset, and (3) onset of symptoms within seven days prior to inclusion. Exclusion criteria were as follows: (1) transient ischemic attack, (2) history of previous stroke, (3) traumatic intracerebral hemorrhage, and (4) non-Korean patients. Written informed consent was obtained from all patients, and the study protocol was approved by the Pusan National University Yangsan Hospital Institutional Review Board (05-2012-057).

For this study, we selected patients with available functional outcome data at 6 months post-stroke. These selected patients constituted the entire sample for our study. Subjects were categorized by sex into male and female groups and by the degree of initial motor function into mild, moderate, severe, and very severe subgroups for further analysis.

### 2.2. Demographics and Height

Demographic data included in this study were age, sex, height, weight, and BMI. Height was measured using an automated stadiometer. For patients who could stand, height measurements were taken using a standard upright position. For patients unable to stand, height was measured in a supine position on a flat surface, ensuring the alignment of heels, buttocks, back, and head, and avoiding knee flexion. Height was measured from the crown of the head to the tips of the heels using a tape measure.

In this study, we attempted to analyze the relationship between height and functional outcomes after stroke. Because height varies depending on race, sex, and age, comparing height using absolute values is inappropriate. Therefore, we categorized height based on quartiles: the first quartile (Q1) corresponds to the 25th percentile, the second quartile (Q2) to the median, and the third quartile (Q3) to the 75th percentile. Using this method, we divided the population into three groups: those below Q1 (25%) were classified as the short group, those between Q1 and Q3 (50%) as the middle group, and those above Q3 (25%) as the tall group.

### 2.3. Initial Motor Function and Functional Outcomes at Six Months

Functional outcome evaluations in the KOSCO study were performed face-to-face in nine representative hospitals. All assessments were performed by qualified evaluators who were licensed occupational or physical therapists and had completed the standard training program provided by the KOSCO study.

The Fugl–Meyer Assessment (FMA) score was used to assess initial motor function in patients with stroke at 7 days post-stroke. The FMA evaluates motor impairment based on the Brunnstrom recovery stages, with scores ranging from 0 (complete paresis) to 100 (normal strength) [25,26]. Additionally, patients were divided into four subgroups based on 7-day FMA scores: <36 (very severe), 36–55 (severe), 56–79 (moderate), and >79 (mild) [27].

The Functional Ambulatory Category (FAC), modified Rankin Scale (mRS), and Korean version of the Modified Barthel Index (K-MBI) were used to assess functional outcomes at 6 months post-stroke. The FAC assesses gait function on a scale from 0 to 5 [28]. The mRS evaluates disability severity on a scale from 0 (no symptoms) to 6 (death) [29]. The K-MBI measures ADLs on a scale from 0 to 100, consisting of 10 ADL items [30,31].

### 2.4. Statistical Analysis

Height was divided into three groups (short, middle, and tall), and the mean values of demographic data for each height category were analyzed using one-way ANOVA and Chi-square tests.

Differences in FAC, mRS, and K-MBI values 6 months post-stroke were analyzed among the three height groups. Subgroup analysis was conducted by dividing patients into four groups based on the FMA score. Differences in the mean values of FAC, mRS, and K-MBI for each height category were analyzed using Analysis of Covariance (ANCOVA) to adjust for confounding variables. *p*-values < 0.05 were considered statistically significant. All statistical analyses were conducted using SPSS version 27.0 (SPSS, Chicago, IL, USA).

## 3. Results

### 3.1. Demographic Characteristics of Patients Based on Height

The baseline KOSCO data included 7858 patients with stroke. After excluding patients without outcome data (FAC, mRS, and K-MBI) at 6 months post-stroke, 5296 initial patients with stroke (3121 male and 2175 female) were selected (Table 1). When patients were divided into three groups based on height, the average height was 151.67 ± 4.55 cm for the short group, 163.00 ± 3.57 cm for the middle group, and 173.52 ± 3.59 cm for the tall group. The average age was 70.18 ± 11.03 years for the short group, 63.62 ± 12.28 years for the middle group, and 58.29 ± 13.24 years for the tall group. Age decreased significantly with increasing height (*p* < 0.05). The average BMI values were 23.47 ± 3.58 kg/m^2^ for the short group, 23.57 ± 3.15 kg/m^2^ for the middle group, and 24.06 ± 3.14 kg/m^2^ for the tall group, increasing significantly with height (*p* < 0.05). In male and female patients, the age decreased significantly, and in male patients, the BMI increased significantly, as height increased (*p* < 0.05). Other demographic data are presented in Table 1.

### 3.2. Functional Outcomes at Six Months According to Height

Functional outcomes at 6 months post-stroke were analyzed after adjusting for age and BMI. When age was adjusted to 63.7 years and BMI to 23.7 kg/m^2^, functional outcomes based on height classification presented values as estimated mean ± standard error. The mRS score was 1.88 ± 0.043 for the short group, 1.46 ± 0.030 for the middle group, and 1.39 ± 0.040 for the tall group (*p* < 0.05). The FAC score was 3.74 ± 0.045 for the short group, 4.16 ± 0.032 for the middle group, and 4.23 ± 0.042 for the tall group (*p* < 0.05). The K-MBI score was 82.83 ± 0.748 for the short group, 87.06 ± 0.525 for the middle group, and 85.29 ± 0.700 for the tall group (*p* < 0.05). The mRS, FAC, and K-MBI scores of the short-height group exhibited a statistical difference from the other height groups. The short-height group had higher mRS scores, lower FAC scores, and lower K-MBI scores than the other height groups (*p* < 0.05) (Table 2). Both male and female patients exhibited the same trend in mRS and FAC scores as the total patient population (*p* < 0.05). However, no significant difference was observed in the K-MBI scores of male and female patients (Table 2).

### 3.3. Subgroup Analysis of Patients According to the FMA Score

Subgroups were created based on the 7-day FMA score, and the relationship between height and functional outcomes at 6 months post-stroke was analyzed. Of the 5296 patients with stroke, 3720 had mild stroke (FMA > 79), 423 had moderate stroke (FMA 56–79), 250 had severe stroke (FMA 36–55), and 901 had very severe stroke (FMA < 36).

In the group with mild FMA values, after adjusting age and BMI to 63.0 years and 23.8 kg/m^2^, the mRS score was 1.72 ± 0.048 for the short group, 1.43 ± 0.035 for the middle group, and 1.13 ± 0.045 for the tall group. The FAC score was 3.91 ± 0.049 for the short group, 4.19 ± 0.036 for the middle group, and 4.49 ± 0.046 for the tall group (*p* < 0.05). The K-MBI score was 93.72 ± 0.457 for the short group, 96.01 ± 0.333 for the middle group, and 95.72 ± 0.431 for the tall group (*p* < 0.05). The group with mild FMA values exhibited higher mRS scores, lower FAC scores, and lower K-MBI scores in the short-height group (*p* < 0.05) (Table 3). The group with moderate FMA values exhibited the same trend in mRS, FAC, and K-MBI scores as the mild FMA group (*p* < 0.05). The group with severe FMA values exhibited the same trend in mRS and K-MBI scores as the mild FMA group (*p* < 0.05).

The group with very severe FMA values exhibited lower mRS scores and higher FAC scores in the middle-height group (*p* < 0.05). The K-MBI scores of the very severe FMA group showed no statistical difference (Table 3).

## 4. Discussion

This study demonstrated that the short-height group had worse outcomes in terms of disability, gait function, and ADLs at 6 months post-stroke.

Previous studies have identified various physical factors influencing functional outcomes after a stroke, such as age, BMI, and sex. However, few studies have examined the influence of height, one of the leading and easily measurable anthropometric factors, on functional outcomes post-stroke. In the previous study, no correlation has been reported between height and disability, as assessed by mRS, at discharge [12]. No study has analyzed the association between height and functional outcomes post-stroke beyond these findings. As one previous study evaluated outcomes only in terms of disability, this study further analyzed the influence of height on disability, gait function, and ADLs post-stroke by categorizing height into three groups: short, middle, and tall. Additionally, analyses were conducted separately for male and female groups due to their different average heights.

Height was categorized into three groups based on specific criteria. Medically, short stature is defined as height two standard deviations (SD) below the population mean [32], and tall stature as height two SD above the population mean [33]. However, these definitions often refer to pathological shortness or tallness due to hormonal or genetic abnormalities [34,35]. Additionally, there cannot be an absolute standard for height because the average height varies depending on race, age, sex, etc. Given the mixed ages and sexes of our study’s populations, it was determined that height could not be classified into “tall” or “short” based solely on absolute values. Considering our substantial sample size of 5296 subjects, which ensures representativeness, we grouped the subjects into quartiles: the bottom 25th percentile as the short group, the 25th to 75th percentiles as the middle group, and the top 25th percentile as the tall group. Although our study was conducted with patients of a single race, it is suggested that the use of relative, rather than absolute, height values allow the findings to be potentially applicable to other racial groups as well.

We hypothesized that a patient’s height might differentially affect their recovery depending on initial motor impairment. For example, patients with very severe motor impairment focus on a passive range of motion rehabilitation, whereas those with mild motor impairment focus on balance and gait training. Thus, we thought that patient height might have a different impact on functional outcomes in chronic stroke patients based on their initial motor function. We divided patients into mild, moderate, severe, and very severe motor impairment groups based on the FMA score 7 days post-stroke, and investigated the relationship between height and functional outcomes in each subgroup.

The results showed that the degree of disability at 6 months post-stroke was more severe in the short-height group, with this trend being consistent among both male and female groups. In the subgroup analysis, disability at 6 months post-stroke was more severe in the short-height group, regardless of initial motor function. However, in the very severe FMA group, the middle-height group had better outcomes. The very severe FMA group had a severe initial disability that makes it difficult to achieve proportional recovery [36]. This may have contributed to their different outcome compared to other FMA groups.

Latisha et al.’s study on 881 patients with ischemic stroke found no association between height and disability at discharge [12]. However, our study found that the short-height group had a more severe disability. Latisha et al. categorized disability into two groups based on mRS—disabled (2–6 points) and non-disabled (0–1 points)—and compared median height between the two groups. In contrast, we divided height into three groups and analyzed disability differences within each group. Therefore, compared with previous studies, our results better reflect the degree of disability relative to height.

The short-height group also had worse gait function at 6 months post-stroke, which was consistent for both male and female groups. This trend remained in subgroup analysis, except for the very severe and severe FMA groups. Gait disorders occur in approximately 80% of patients with stroke [37], with paralysis leading to poor posture and balance [38]. Postural distortion and poor balance result in poor gait function in patients with stroke [39]. Therefore, balance regulation is important for gait, and the increase in COG associated with tall height may lead to a decline in balance, potentially resulting in poorer gait function in taller stroke patients [21]. However, no studies have analyzed the relationship between height and balance or gait function in post-stroke patients. In healthy young adults, taller height has been associated with poorer balance, expressed as a medial–lateral displacement and displacement area and sway velocity [40]. Conversely, in a study of healthy young females, taller females had better balance outcomes than shorter females when subjected to an external destabilizing stimulus [41]. Furthermore, no significant sex differences have been observed in the degree of body imbalance caused by an external destabilizing stimulus [42]. Previous studies have shown inconsistent results regarding height and balance. Contrary to conventional beliefs, our study found that the short-height group exhibited worse gait function after stroke. The results of our study suggested that other height-related factors influence gait function more than overall imbalance, which increases with height due to the higher COG.

The short-height group also had worse ADLs at 6 months post-stroke. There was no statistical significance in both male and female groups. A subgroup analysis showed worse ADLs in the short-height group, except for the very severe FMA group. Dependency in personal ADLs is a common short-term and long-term consequence of stroke [43]. While physical factors influencing ADLs are well-known, no studies have analyzed the relationship between height and ADLs in patients with stroke. Jeong et al.’s study on 64 severely disabled stroke patients found that K-MBI scores at 3 months were influenced by sitting, standing balance, and sit-up ability [44]. Kim et al.’s study on 123 patients with stroke found a strong positive correlation between K-MBI and FAC scores [45]. Many K-MBI items relate to transfer and ambulation, aligning the relationship between height and ADLs with our findings on disability and gait function. However, K-MBI items such as personal hygiene, eating, and bowel and bladder control are not directly influenced by gait function. Therefore, they may be affected by other height-related factors.

Several physiological theories have been studied regarding how height can affect medical conditions. The shorter arterial lengths in shorter individuals lead to earlier pressure wave reflections and a greater summation of reflected pressure, increasing hypertension incidence [46]. Hypertension drives microglial polarization, promoting a pro-inflammatory state that weakens brain plasticity and impairs stroke recovery [47]. Shorter individuals also often have reduced beta-cell function and higher insulin resistance, increasing type 2 diabetes mellitus incidence [48]. Inefficient glucose metabolism adversely affects brain function, potentially worsening post-stroke outcomes [49]. However, our study could not determine which physiological theory—whether related to medical characteristics or the physical characteristics of COG, etc.—affects the relationship between height and functional outcomes post-stroke. Therefore, systematic follow-up studies integrating patients’ underlying conditions and various health data are necessary for clarification.

Compared with other studies, this study has several strengths. First, it is a large-scale national multicenter study of 5296 patients with stroke in Korea, providing high statistical significance. Second, while previous studies primarily evaluated stroke outcomes in terms of disability, our research also analyzed gait function and ADLs. Third, there is no absolute standard for height, as average height varies by race, sex, and age. Unlike previous studies that simply compare absolute height values, our study analyzed height on a relative basis using percentiles. Fourth, through a sub-analysis not conducted in previous studies, we examined the impact of height according to initial motor impairment and presented the results.

However, this study has several limitations. First, we analyzed functional outcomes only at 6 months post-stroke, indicating a need for long-term analysis to understand the enduring impacts of height on recovery. Second, although the study was based on a large population, it did not account for initial stroke severity (e.g., national institutes of health stroke scale (NIHSS) scores), which affects the recovery of stroke patients [50]. Future studies should adjust for patient factors such as age and obesity, as well as other factors that may affect recovery. Third, this study revealed trends in the relationship between height and disability, gait function, and ADLs after a stroke. However, a physiological theory to support these findings is lacking. Therefore, systematic follow-up studies are needed for clarification.

## 5. Conclusions

Our study indicates that the short-height group exhibited worse outcomes in terms of disability, gait function, and ADLs at 6 months post-stroke. When male and female groups were analyzed separately, the short-height group consistently showed worse outcomes in terms of disability and gait function. Furthermore, in the subgroup analysis based on initial motor function, the short-height group demonstrated worse functional outcomes, except for those with a very severe initial impairment. There is a notable association between height and functional outcomes such as disability, gait function, and ADLs in the chronic phase of stroke, which may aid in predicting functional recovery. However, the underlying physiological mechanisms remain unclear. Further research is necessary to draw more definitive conclusions.

## Figures and Tables

**Table 1:** Demographic characteristics of patients based on height.

Total (*n* = 5296)	Short (*n* = 1317)	Middle (*n* = 2495)	Tall (*n* = 1484)	*p*-Value
Age (years)	70.18 ± 11.03	63.62 ± 12.28	58.29 ± 13.24	<0.001 *
Height (cm)	151.67 ± 4.55	163.00 ± 3.57	173.52 ± 3.59	<0.001 *
Weight (kg)	54.09 ± 8.60	62.72 ± 8.90	72.58 ± 10.10	<0.001 *
BMI (kg/m^2^)	23.47 ± 3.58	23.57 ± 3.15	24.06 ± 3.14	<0.001 *
**Male (*n* = 3121)**	**Short (*n* = 773)**	**Middle (*n* = 1394)**	**Tall (*n* = 954)**	***p*-Value**
Age (years)	66.43 ± 10.77	62.78 ± 11.89	56.94 ± 13.38	<0.001 *
Height (cm)	160.09 ± 3.68	168.03 ± 2.02	175.33 ± 3.25	<0.001 *
Weight (kg)	61.12 ± 8.38	67.06 ± 8.68	74.55 ± 10.33	<0.001 *
BMI (kg/m^2^)	23.81 ± 3.07	23.75 ± 3.02	24.23 ± 3.21	0.001 *
**Male (*n* = 2175)**	**Short (*n* = 529)**	**Middle (*n* = 1010)**	**Tall (*n* = 636)**	***p*-Value**
Age (years)	72.67 ± 10.24	66.41 ± 11.91	61.21 ± 14.54	<0.001 *
Height (cm)	147.91 ± 4.80	155.57 ± 1.99	162.47 ± 3.03	<0.001 *
Weight (kg)	51.36 ± 7.79	56.86 ± 8.43	61.30 ± 9.55	<0.001 *
BMI (kg/m^2^)	23.40 ± 3.57	23.48 ± 3.43	23.15 ± 3.44	0.169

BMI: body mass index, * indicates *p* < 0.05.

**Table 2:** Functional outcomes at six months according to height.

Total (*n* = 5296)	Short (*n* = 1317)	Middle (*n* = 2495)	Tall (*n* = 1484)	*p*-Value
mRS_6m	1.88 ^a^ ± 0.043	1.46 ^b^ ± 0.030	1.39 ^b^ ± 0.040	<0.001 *
FAC_6m	3.74 ^a^ ± 0.045	4.16 ^b^ ± 0.032	4.23 ^b^ ± 0.042	<0.001 *
K-MBI_6m	82.83 ^a^ ± 0.748	87.06 ^ab^ ± 0.525	85.29 ^b^ ± 0.700	<0.001 *
※ Adjusted age = 63.7 years, BMI = 23.7 kg/m^2^				
**Male (*n* = 3121)**	**Short (*n* = 773)**	**Middle (*n* = 1394)**	**Tall (*n* = 954)**	***p*-Value**
mRS_6m	1.53 ^a^ ± 0.053	1.30 ^b^ ± 0.039	1.35 ^b^ ± 0.048	<0.001 *
FAC_6m	4.06 ^a^ ± 0.054	4.31 ^b^ ± 0.039	4.25 ^b^ ± 0.055	<0.001 *
K-MBI_6m	89.50 ± 0.748	89.11 ± 0.640	86.91 ± 0.792	0.052
※ Adjusted age = 61.9 years, BMI = 23.9 kg/m^2^				
**Female (*n* = 2175)**	**Short (*n* = 529)**	**Middle (*n* = 1010)**	**Tall (*n* = 636)**	***p*-Value**
mRS_6m	2.06 ^a^ ± 0.071	1.74 ^b^ ± 0.050	1.64 ^b^ ± 0.064	<0.001 *
FAC_6m	3.58 ^a^ ± 0.003	3.90 ^b^ ± 0.055	3.98 ^b^ ± 0.071	<0.001 *
K-MBI_6m	81.76 ± 1.299	82.34 ± 0.908	78.83 ± 1.176	0.057
※ Adjusted age = 66.4 years, BMI = 23.4 kg/m^2^				

The values of this table are estimated values; estimated mean (em mean) ± standard error (se); mRS, modified Rankin score; FAC, functional ambulation category; K-MBI, Korean version of the modified Barthel index; ^a^, ^b^, ^ab^ indicate a difference between different characters; * indicates a significant difference (*p* < 0.05).

**Table 3:** Functional outcomes at six months according to FMA.

Mild	Short (*n* = 950)	Middle (*n* = 1705)	Tall (*n* = 1065)	*p*-Value
mRS_6m	1.72 ^a^ ± 0.048	1.43 ^b^ ± 0.035	1.13 ^ab^ ± 0.045	<0.001 *
FAC_6m	3.91 ^a^ ± 0.049	4.19 ^b^ ± 0.036	4.49 ^ab^ ± 0.046	<0.001 *
K-MBI_6m	93.72 ^a^ ± 0.457	96.01 ^b^ ± 0.333	95.72 ^b^ ± 0.431	<0.001 *
※ Adjusted age = 63.0 years, BMI = 23.8 kg/m^2^				
**Moderate**	**Short (*n* = 118)**	**Middle (*n* = 181)**	**Tall (*n* = 124)**	***p*-Value**
mRS_6m	2.09 ^a^ ± 0.147	1.53 ^b^ ± 0.113	1.47 ^b^ ± 0.143	0.004 *
FAC_6m	3.45 ^a^ ± 0.153	4.18 ^b^ ± 0.118	4.25 ^b^ ± 0.149	<0.001 *
K-MBI_6m	78.13 ^a^ ± 2.269	85.08 ^b^ ± 1.751	81.64 ^ab^ ± 2.217	0.048 *
※ Adjusted age = 66.3 years, BMI = 23.6 kg/m^2^				
**Severe**	**Short (*n* = 64)**	**Middle (*n* = 115)**	**Tall (*n* = 71)**	***p*-Value**
mRS_6m	2.26 ^a^ ± 0.186	1.44 ^b^ ± 0.135	1.49 ^b^ ± 0.173	0.002 *
FAC_6m	3.50 ^a^ ± 0.209	4.14 ^b^ ± 0.151	3.98 ^ab^ ± 0.194	0.052
K-MBI_6m	64.39 ^a^ ± 3.618	77.17 ^b^ ± 2.616	77.34 ^b^ ± 3.356	0.011 *
※ Adjusted age = 63.9 years, BMI = 23.5 kg/m^2^				
**Very severe**	**Short (*n* = 235)**	**Middle (*n* = 413)**	**Tall (*n* = 253)**	***p*-Value**
mRS_6m	2.21 ^a^ ± 0.115	1.57 ^b^ ± 0.083	2.37 ^a^ ± 0.110	<0.001 *
FAC_6m	3.35 ^a^ ± 0.127	3.99 ^b^ ± 0.091	3.24 ^a^ ± 0.121	<0.001 *
K-MBI_6m	48.63 ± 2.209	51.26 ± 1.580	48.12 ± 2.103	0.398
※ Adjusted age = 63.7 years, BMI = 23.7 kg/m^2^				

The values of this table are estimated values; estimated mean (em mean) ± standard error (se); mRS, modified Rankin score; FAC, functional ambulation category; K-MBI, Korean version of the modified Barthel index; ^a^, ^b^, ^ab^ indicate a difference between different characters; * indicates a significant difference (*p* < 0.05).

## Data Availability

The authors do not have permission to share the data.
